# Awakening p53 in senescent cells using nutlin-3

**DOI:** 10.18632/aging.100094

**Published:** 2009-10-13

**Authors:** Thaddeus T. Schug

**Affiliations:** Laboratory of Signal Transduction, National Institute of Environmental Health Sciences, National Institutes of Health, Research Triangle Park, NC 27709

**Keywords:** Senescence, p53, tumor suppression, nutlin-3

The p53 tumor suppressor protein is arguably the most important guardian of the mammalian genome. p53 promotes longevity by reducing somatic mutations or the survival and proliferation of mutant cells. Under normal conditions, this proteins role is inconsequential because of the rapidity of its degradation. But stress signals of almost any form halt the degradation of p53, unleashing an active protein that triggers transient cell cycle arrest, apoptosis, or cellular senescence [1]. Both apoptosis and senescence are potent tumor suppressor mechanisms that irreversibly prevent tumorgenesis [2]. However, both processes also deplete tissues of proliferation-competent progenitor cells, which is a characteristic associated with degenerative aging. Therefore mammalian survival paradoxically lies at the mercy of p53, which oversees a tight balance between tumor suppression and long-term cell proliferation.

Almost all human cancers contain impairments in the p53 signaling pathway [3]. Roughly one half of cancers have inactivating mutations of p53, while the remaining half retain function but have miscued activation, degradation or cell-cycling. Intense focus on understanding p53 regulation at the molecular level has led to interest in isolating and developing small-molecule targets of p53 activity to treat various forms of cancer. Nutlins are cis-imidazoline analogs, which inhibit the interaction between MDM2 (murine double minute 2) and p53, and were discovered by screening a chemical library done by Vassilev and colleagues [4]. MDM2 negatively regulates p53 by binding to and transporting the protein to the cytoplasm where it attaches to an E3 ubiquitin ligase and becomes subject to proteosomal degradation.  Nutlins prevent p53-MDM2 interaction, and thus induce expression of p53-regulated genes, which exhibit potent anti-proliferative activities (Figure 1A).

Senescence is a complex genetic program and a cell fate decision that establishes permanent growth arrest, which is a theurapeutic goal in cancer treatment. Although there is no direct link between cellular senescence and aging, a correlation does exist, as the number of senescent cells increases in mammals as they age [5]. Activation of p53 is crucial for initiating and maintaining senescence in most cell types. However, the pro-apoptotic arm of the p53 signaling pathway in some senescent cells is dysfunctional [6,7]. In this issue of Aging, Huang et al. explore p53 transcriptional activity and apoptosis in senescent human lung fibroblasts using nutlin-3. Because nutlin-3 is not genotoxic and does not cause p53 phosphorylation or deacetylation, it effectively allows investigation of downstream p53 signaling in senescent cells.

Senescent cells are characterized by highly compacted heterochromatin, termed "senescence-associated hetero-chromatin foci", or SAHF [8]. These SAHF are thought to lack sites of active gene transcription, as bulk DNA is less accessible to the loading of necessary transcription factors [9]. Huang et al. observed morphological traits typical of senescence in human fibroblast cells (WI-38), however, they also noted that basal p53 expression levels and target gene activity was normal, or even slightly higher. In an attempt to induce p53 transcription without affecting upstream signaling, the investigators exposed cells to nutlin-3. Although protein levels remain consistent in both early passage and senescent WI-38 cells, transcriptional activity of p53 was attenuated upon nutlin-3 treatment. Moreover, the combined treatment of nutlin-3, together with the DNA damaging compound doxorubicin, boosted p53 levels and restored normal transcriptional activity.

**Figure 1.**
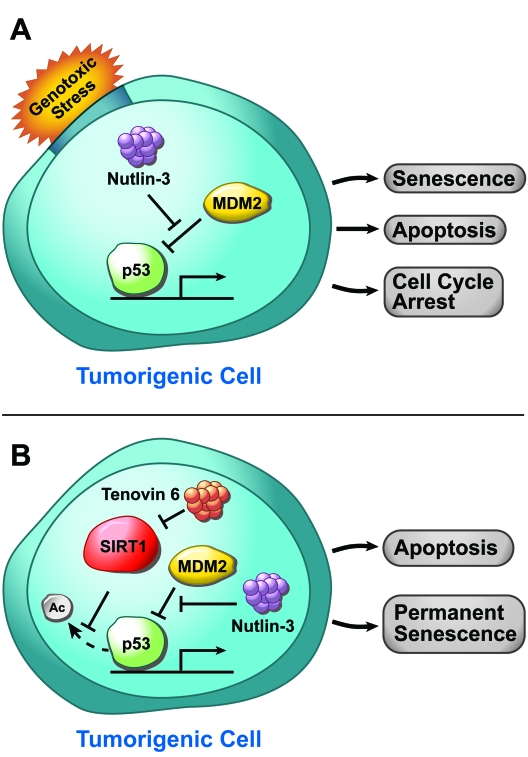
The p53 signaling pathway in response to genotoxic stress in tumorogenic cells. (**A**) The MDM2 inhibitor nutln-3 increases p53-mediated senescence, apoptosis, and cell cycle arrest. (**B**) The SIRT1 small-molecule inhibitor Tenovin 6 works in tandem with nutlin-3 to induce apoptosis or permanent senescence in cancer cells.

Huang et al. conclude that the decline in p53 functionality in senescent cells is due to changes in upstream signaling that ultimately leads to destabilization of p53 and abrogated signaling.  They point to the possibility that the pro-apoptotic functions of p53 may be redundant in senescent cells, which have already lost their ability to proliferate and become cancerous.  These findings add to a recent publication by this group revealing that nutlin-3 can induce a senescence-like state in several epithelial cancer cell lines [10], but it is reversible and cells resume proliferation upon drug removal and normalization of p53 signaling. Perhaps in senescent cells p53 is at a balance, coordinating between DNA damage signaling and apoptosis. Further studies will be important to help identify and optimize therapeutic targets used to treat tumor suppression without the downsides associated with continual drug treatment or accelerating aging.

The study by Huang and coworkers highlights several important issues in cancer therapy development. In addition to p53-mediated apoptosis, nutlins may induce and maintain a permanent dormancy in cancer cells. The ability for nutlin-3 to activate p53 without the genotoxic stresses incurred by most chemotherapeutic agents is of tremendous clinical importance. It may be of value to note that acetylation of p53 also inhibits p53-MDM2 interactions [11,12]. Several recent reports have identified the longevity gene, SIRT1, as key regulator of p53 acetylation [13-15]. Use of small molecule inhibitors of SIRT1, such as Tenovin 6, in tandem with nutlins, may provide a double hit on the p53 pathway and push the balance toward acetylated and activated p53 protein levels sufficient enough to induce apoptosis in cancer cells (Figure 1B) [16].

## References

[1] Collado M, Blasco MA, Serrano M (2007). Cellular senescence in cancer and aging. Cell.

[2] Junttila MR, Evan GI (2009). p53 - a Jack of all trades but master of none. Nat Rev Cancer.

[3] Kumamoto K, Spillare EA, Fujita K, Horikawa I, Yamashita T, Appella E, Nagashima M, Takenoshita S, Yokota J, Harris CC (2008). Nutlin-3a activates p53 to both down-regulate inhibitor of growth 2 and up-regulate mir-34a, mir-34b, and mir-34c expression, and induce senescence. Cancer Res.

[4] Vassilev LT, Vu BT, Graves B, Carvajal D, Podlaski F, Filipovic Z, Kong N, Kammlott U, Lukacs C, Klein C (2004). In vivo activation of the p53 pathway by small-molecule antagonists of MDM2. Science.

[5] Lombard DB, Chua KF, Mostoslavsky R, Franco S, Gostissa M, Alt FW (2005). DNA repair, genome stability, and aging. Cell.

[6] Seluanov A, Gorbunova V, Falcovitz A, Sigal A, Milyavsky M, Zurer I, Shohat G, Goldfinger N, Rotter V (2001). Change of the death pathway in senescent human fibroblasts in response to DNA damage is caused by an inability to stabilize p53. Mol Cell Biol.

[7] Yeo EJ, Hwang YC, Kang CM, Choy HE, Park SC (2000). Reduction of UV-induced cell death in the human senescent fibroblasts. Mol Cells.

[8] Narita M, Nunez S, Heard E, Narita M, Lin AW, Hearn SA, Spector DL, Hannon GJ, Lowe SW (2003). Rb-mediated heterochromatin formation and silencing of E2F target genes during cellular senescence. Cell.

[9] Schulz L, Tyler J (2005). Heterochromatin focuses on senescence. Mol Cell.

[10] Huang B, Deo D, Xia M, Vassilev LT (2009). Pharmacologic p53 activation blocks cell cycle progression but fails to induce senescence in epithelial cancer cells. Mol Cancer Res.

[11] Brooks CL, Gu W (2006). p53 ubiquitination: Mdm2 and beyond. Mol Cell.

[12] Solomon JM, Pasupuleti R, Xu L, McDonagh T, Curtis R, DiStefano PS, Huber LJ (2006). Inhibition of SIRT1 catalytic activity increases p53 acetylation but does not alter cell survival following DNA damage. Mol Cell Biol.

[13] Cheng HL, Mostoslavsky R, Saito S, Manis JP, Gu Y, Patel P, Bronson R, Appella E, Alt FW, Chua KF (2003). Developmental defects and p53 hyperacetylation in Sir2 homolog (SIRT1)-deficient mice. Proc Natl Acad Sci U S A.

[14] Tang Y, Zhao W, Chen Y, Zhao Y, Gu W (2008). Acetylation is indispensable for p53 activation. Cell.

[15] Chua KF, Mostoslavsky R, Lombard DB, Pang WW, Saito S, Franco S, Kaushal D, Cheng HL, Fischer MR, Stokes N (2005). Mammalian SIRT1 limits replicative life span in response to chronic genotoxic stress. Cell Metab.

[16] van Leeuwen I, Lain S (2009). Sirtuins and p53. Adv Cancer Res.

